# 1021. The post-sign-off events of Infectious Diseases (ID) consultation and the incidence of non-adherence to ID consultation during the post-sign-off period: A retrospective cohort study

**DOI:** 10.1093/ofid/ofac492.862

**Published:** 2022-12-15

**Authors:** Hitoshi Honda, Akane Takamatsu

**Affiliations:** Fujita Health University School of Medicine, Toyoake, Aichi, Japan; Tokyo Metropolitan Tama Medical Center, Fuchu, Tokyo, Japan

## Abstract

**Background:**

Infectious Diseases (ID) consultation has contributed to improving the quality of care in inpatient settings. ID physicians traditionally examine patients and provide the final treatment recommendations (i.e., sign-off ID consultation). However, patients may acquire antimicrobial-associated adverse drug events (AAADE) and healthcare-associated infections (HAI) after the sign-off ID consultation. Moreover, it is unclear how these events that occurred during the post-sign-off period were managed. The study aims to scrutinize the post-sign-off events and the current epidemiology of non-adherence to ID recommendations during the post-sign-off period.

**Methods:**

The retrospective cohort study was performed at a Japanese tertiary care center. Among all ID consultation cases from January to December 2019, those with general ID consultation with treatment recommendations given for the definitive or suspected infectious diseases were included for analysis. The instances of post-sign-off events included AAADE, HAI, and treatment failure of established infections. Non-adherence to ID consultation was defined as the failure of the primary care team to complete ID final recommendations. The incidence of the post-sign-off events and outcomes associated with nonadherence to ID consultations were investigated.

**Results:**

Among 762 ID consultations that occurred, 367 cases were included for analysis. The incidence of the post-sign-off events was 10.9% (40/367), and AAADEs accounted for 62.5% (25/40) of cases. Non-adherence to ID consultation occurred in 105 (28.6%) cases, and prolonged antimicrobial therapy beyond ID consultation was the most common reason for non-adherence. The primary care team modified antimicrobial therapy in approximately two-thirds of cases (71/105) without ID re-consultation. The length of hospital stay was longer in patients with non-adherence to ID consultation than in those with adherence (36 versus 29 days; p=0.002).

**Conclusion:**

The post-sign-off events of ID consultation commonly occur, leading to nonadherence to ID consultation during the post-sign-off period. Because nonadherence may be associated with worse clinical outcomes, attention after the sign-off ID consultation is needed to ensure patient safety.

**Disclosures:**

**All Authors**: No reported disclosures.

